# Tetrandrine Induces Mitochondria-Mediated Apoptosis in Human Gastric Cancer BGC-823 Cells

**DOI:** 10.1371/journal.pone.0076486

**Published:** 2013-10-01

**Authors:** Rong Qin, Huiling Shen, Yuan Cao, Yue Fang, Hao Li, Qiaoyun Chen, Wenlin Xu

**Affiliations:** 1 Department of Oncology, The Affiliated People’s Hospital, Jiangsu University, Zhenjiang, Jiangsu, China; 2 The Affiliated Fourth People’s Hospital, Jiangsu University, Zhenjiang, Jiangsu, China; Thomas Jefferson University, United States of America

## Abstract

Tetrandrine, a bis-benzylisoquinoline alkaloid isolated from the dried root of Hang-Fang-Chi ( *Stephania* *tetrandra* S. Moore), has been reported to possess anti-cancer effects on many tumors. In this study, we investigated tetrandrine-induced apoptosis on human gastric cancer BGC-823 cells *in vitro* and *in vivo*. The results showed that tetrandrine significantly inhibited cell viability in a dose- and time-dependent manner and induced apoptosis. It increased the apoptosis; upregulation of Bax, Bak, and Bad; and downregulation of Bcl-2 and Bcl-xl in BGC-823 cells. Moreover, tetrandrine increased the activation of caspase-3 and -9, release of cytochrome *c*, and upregulation of apaf-1, suggesting that tetrandrine-induced apoptosis was related to the mitochondrial pathway. Meanwhile, pretreatment with the pan-caspase inhibitor z-VAD-fmk in BGC-823 cells reduced tetrandrine-induced apoptosis by blocking activation of caspases. Furthermore, tetrandrine effectively inhibited tumor growth via apoptosis induction, which was verified by immunohistochemical analysis in a nude mouse xenograft model. Taken together, we concluded that tetrandrine significantly inhibited the proliferation of gastric cancer BGC-823 cells through mitochondria-dependent apoptosis, which may play a promising role in gastric cancer therapy.

## Introduction

In China, gastric cancer, one of the most common malignant tumors, resulting from an accumulation of genetic and epigenetic events, still has a high incidence and mortality rate [1-3]. There are several factors causing the onset of gastric cancer, such as *Helicobacter Pylori* infection, diet, tobacco use, and so on [4]. Depending on the circumstances, most patients with gastric cancer need surgery, chemotherapy, and/or chemoradiotherapy [5]. Previous studies have revealed that gastric cancer is a complex genetic disease related to oncogenes or tumor suppressors [6].

Tetrandrine (C_38_H_42_N_2_O_6_, MW 622.730) is a bis-benzylisoquinoline alkaloid isolated from the dried root of Hang-Fang-Chi ( *Stephania* *tetrandra* S. Moore). Recently, the diverse biological activities of tetrandrine have been studied intensively because of its wide use in arthritis, arrhythmia, inflammation, silicosis, various types of tumors, and reverse multi-drug resistance [7,8]. It is reported that tetrandrine has significant effects on tumors including slowing tumor growth and increasing animal survival time and survival rate [9-12]. Tetrandrine causes a G1 cell-cycle blockade and induces apoptosis in various cell types. However, the precise mechanisms by which tetrandrine initiates apoptosis and inhibits cell growth in gastric tumor cells remain unclear [13]. It is reported that the codelivery of paclitaxel (Ptx) and tetrandrine by nanoparticles can efficiently enhance the cytotoxicity of Ptx by sequential inhibition of the ROS-dependent Akt pathway and activation of apoptotic pathways based on oxidation therapy against gastric cancer [14]. However, as a therapeutic agent, Ptx inevitably has severe side effects owing to its nonspecific toxic effects and special solvents, and tumor cells may become more resistant to Ptx-induced apoptosis [15-17]. A previous study has shown that tetrandrine has a synergistic effect with chemotherapeutic agents on apoptosis of gastric cancer cell lines [18]. Furthermore, a derivative (H1) of tetrandrine exerts good anti-multidrug resistance activity by initiating the intrinsic apoptosis pathway and inhibiting the activation of Erk1/2 and Akt1/2 [19]. These findings suggest that tetrandrine might replace Ptx as the first line chemotherapy for gastric cancer.

Since tetrandrine has great potential in anti-cancer therapy, it is important to study it systematically to understand its mechanism. Therefore, in this study, we investigated the possible mechanisms of tetrandrine on human gastric cancer cells *in vitro* and *in vivo*.

## Materials and Methods

### Materials and reagents

Tetrandrine was obtained from Jiangxi Yintao Pharmaceutical Development Company (Jiangxi, China). Antibodies were obtained from the following sources: antibodies against Bcl-2, Bax, Bcl-xl, Caspase-3, -8,-9, cytochrome *c*, Apaf-1, and β-actin were from Santa Cruz Biotechnology (Santa Cruz, CA, USA); anti-Bad and Bak were from Bioworld Technology (St. Louis Park, MN, USA). The Trizol reagent kit was purchased from Invitrogen (Grand Island, NY, USA). MTT (3-(4, 5-dimethylthiazol-2-yl)-2, 5-diphenyltetrazolium bromide) was purchased from Sigma-Aldrich (St. Louis, MO, USA).

### Cell culture

The human gastric cancer cell line BGC-823 was purchased from Shanghai Cell Biology, Chinese Academy of Sciences (Shanghai, China). Cells were cultured in Dulbecco’s modified Eagle’s medium (DMEM) (Gibco BRL, Grand Island, NY, USA), supplemented with 10% fetal bovine serum (Hyclone, Logan, UT, USA), 1% penicillin, and 1% streptomycin in a humidified atmosphere of 95% air and 5% CO_2_ at 37 °C.

### Cell viability assay

Cell viability was measured by the MTT assay. In brief, cells were seeded in 96-well plates at a density of 1×10^4^ cells/well. After 36 h, cells were treated with or without tetrandrine at variable concentrations for 24, 48, and 72 h; and phosphate-buffered saline (PBS) served as a negative control. Then, 20 µl of MTT dye solution (0.5 mg/ml, dissolved in PBS and filtered through a 0.2-mm membrane) was added to each well and incubated at 37 °C for 4 h. Thereafter, 150 µl of dimethyl sulfoxide was added to each well, and the plates were incubated at 37 °C for an additional 10 min. Absorbance was measured at 490 nm using a 96-well microplate reader. The IC_50_ value was defined as the concentration of drug that inhibits 50% cell growth compared with the control.

### Western blotting

Gastric cancer cells were washed twice with PBS, and then 100 µl of RIPA Lysis Buffer (Beyotime, China) was added to each well to lyse cells for about 20 min. Next, the mixture was centrifuged at 12,000*g* for 15 min, and the supernatant was collected. Protein concentration was detected by using a BCA Protein Assay Kit (Beyotime, China) according to the manufacturer’s instructions. Total protein was separated by SDS-PAGE on 8%, 10%, and 12% polyacrylamide gels and transferred electrophoretically onto polyvinylidene difluoride (PVDF) membranes (Millipore, Bedford, MA, USA). After blocking for 2 h or overnight with 5% non-fat dry milk (dissolved in Tris-buffered saline-Tween 20 buffer; TBST), membranes were incubated with primary antibodies (1:500-1:1000 dilution) for 2 h at room temperature or overnight at 4 °C and then washed three times with TBST (5 mM Tris-HCl, pH 7.4, 136 mM NaCl, 0.1% Tween 20) before reacting with horseradish peroxidase (HRP)-conjugated secondary antibodies (1:5000-1:10000) for 1 h at room temperature. After washing three times for 10 min each with TBST, the membranes were treated with ECL Western Blotting Detection Reagent.

### Quantitative real-time polymerase chain reaction

Total cellular RNA was isolated using Trizol reagent (Invitrogen) following the manufacture’s protocol and dissolved in DEPC-treated water. The concentration of total RNA was measured by UV absorbance spectroscopy. Reverse transcription was performed using a RevertAid™ First Strand cDNA Synthesis Kit (Fermentas MBI, Waltham, MA, USA) following the manufacturer’s protocol. The newly synthesized cDNA was amplified by polymerase chain reaction (Fermentas). The sequences of each primer used in this study are shown in Table 1. The polymerase chain reaction conditions are listed as follows: 95 °C for 3 min, 95 °C for 5 min, 58 °C for 30 min, and 72 °C for 30 min followed by 40 cycles at 94 °C for 15 s and 60 °C for 1 min. All tests were performed in triplicate.

**Table 1:** Sequences of primers for the genes used in this study.

**Gene**	**Sense primer (5’ to 3’)**	**Antisense primer (5’ to 3’)**
bcl-2	GGATCCAGGATAACGGAGGC	CCAGATAGGCACCCAGGGT
bax	ACCAAGAAGCTGAGCGAGTGT	ACAAACATGGTCACGGTCTGC
bclxl	GGCAGGCGACGAGTTTGA	CCCATCCCGGAAGAGTTCAT
β-actin	TGGCACCCAGCACAATGAA	CTAAGTCATAGTCCGCCTAGAAGCA

### Annexin V-propidium iodide binding assay

In brief, cells were seeded in 6-well plates and treated with different concentrations of tetrandrine for 24 h, and PBS served as a negative control. Then, cells were resuspended in 500 µl of cold binding buffer. Cell suspensions were stained against Annexin-V and propidium iodide (BD Pharmingen^TM^, Becton Dickinson, San Jose, CA, USA) according to the manufacturer’s instructions, and then flow cytometry analysis was performed by a FACS (Coulter, Becton Dickinson).

### Caspase-3 activity assay

Briefly, cells were incubated in a 6-well plate at a concentration of 4×10^5^/well and treated with the indicated concentration of drugs. An equal volume of PBS was used as a negative control. Caspase-3 activity was measured using a caspase-3 activity assay kit (Beyotime, China) following the manufacturer’s instructions. The absorbance was then measured at 405 nm using a microplate reader. All the experiments were carried out in triplicate.

### Ethics statement

Procedures involving animals and their care were conducted in conformity with NIH guidelines (NIH Pub. No.85-23, revised 1996) and were approved by the Animal Care and Use Committee of The Affiliated People’s Hospital, Jiangsu University.

### Animal experiments

Female nude mice (BALB/c nu/nu), 4-6 weeks old, were obtained from the Experimental Animal Center of the Chinese Academy of Science (Shanghai, China) and kept under specific pathogen-free conditions in a biological cabinet at the Laboratory Animal Facility of Jiangsu University. The animals were maintained in a 12-h light-dark cycle. Room temperature maintained at around 20 °C, and humidity was kept at about 50% in a room with a filtered air supply.

### Tetrandrine treatment of subcutaneous BGC-823 tumors in mice

To establish a BALB/c mouse xenograft model of human gastric cancer, BGC-823 cells at a concentration of 5.0×106/150 µl were injected into the right flank of each BALB/c nude mouse. Approximately 10 days later (tumor size reached 120–150 mm3), nude mice were randomly divided into three groups (N = 5) and given the following treatments: control (treated with normal saline), low dose group (treated with 40 mg/kg tetrandrine), and high dose group (treated with 120 mg/kg tetrandrine). BALB/c mice were injected intraperitoneally with tetrandrine (200 µl, 40 mg/kg or 120 mg/kg) or an equal volume of normal saline, once a day for 3 weeks, respectively. The tumor size was measured every 3 days in two perpendicular dimensions with vernier calipers, and the tumor volume (TV) was calculated by the formula: TV = length (mm) × width^2^ (mm^2^) × 0.5. Relative tumor volume (RTV) was calculated according to the following formula: RTV = TV_t_/TV_0_, where TV_0_ is the tumor volume at day 0 and TV_t_ is the tumor volume at a given day t. Meanwhile, the animals were weighed twice per week. At the end of the experiment, tumors were excised and fixed in formalin for further analysis.

### Immunohistochemical analysis

The paraffin-embedded samples excised from BGC-823 nude mice were stained using the following antibodies: Bcl-2 and Bax (Boster), Bcl-xl, activated Caspase-3, and activated Caspase-9 (Santa Cruz) for immunohistochemistry. Images were taken with a fluorescence microscope (Carl Zeiss).

### Statistical analysis

All experiments were performed at least three times, and all data were presented as means ± standard deviation (SD). Statistical differences were determined by one-way ANOVA using SPSS 16.0 software. A P value less than 0.05 was considered to be significant.

## Results

### Cytotoxic effect of tetrandrine on BGC-823 cells

**Figure 1:**
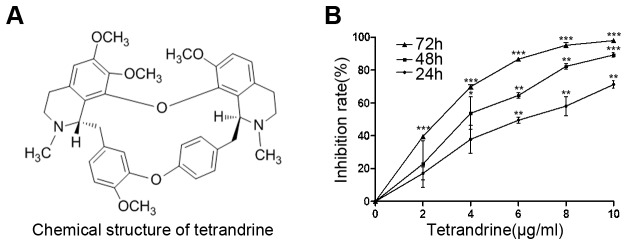
Effect of tetrandrine on the viability of BGC-823 cells. (A) The chemical structure of tetrandrine. (B) Cell proliferation was determined by an MTT assay, and BGC-823 cells were treated with various concentration of tetrandrine at 24, 48 and 72 h, respectively. The inhibition rate of the control group was set to 0. Data are expressed as means ± SD of three independent experiments performed in triplicate. *P < 0.05; **P < 0.01; ***P < 0.001 versus the control group, respectively.

The chemical structure of tetrandrine is shown in Figure 1A. The anti-proliferative effect of tetrandrine was investigated in BGC-823 cells using the MTT assay. As is shown in Figure 1B, the cells were treated for 24, 48, and 72 h with various concentrations of tetrandrine. Tetrandrine was found to significantly inhibit the growth of BGC-823 cells in a dose- and time-dependent manner. BGC-823 cells were significantly inhibited by tetrandrine at 10 μg/ml (*P* < 0.05). The IC_50_ value was 6.104 ± 0.786, 4.471 ± 0.650, and 3.744 ± 0.573 following 24, 48, and 72 h of tetrandrine treatment, respectively. Thus, 6, 8, and 10 μg/ml were determined as the representative concentrations in the following studies.

### Induction of apoptosis by tetrandrine

**Figure 2:**
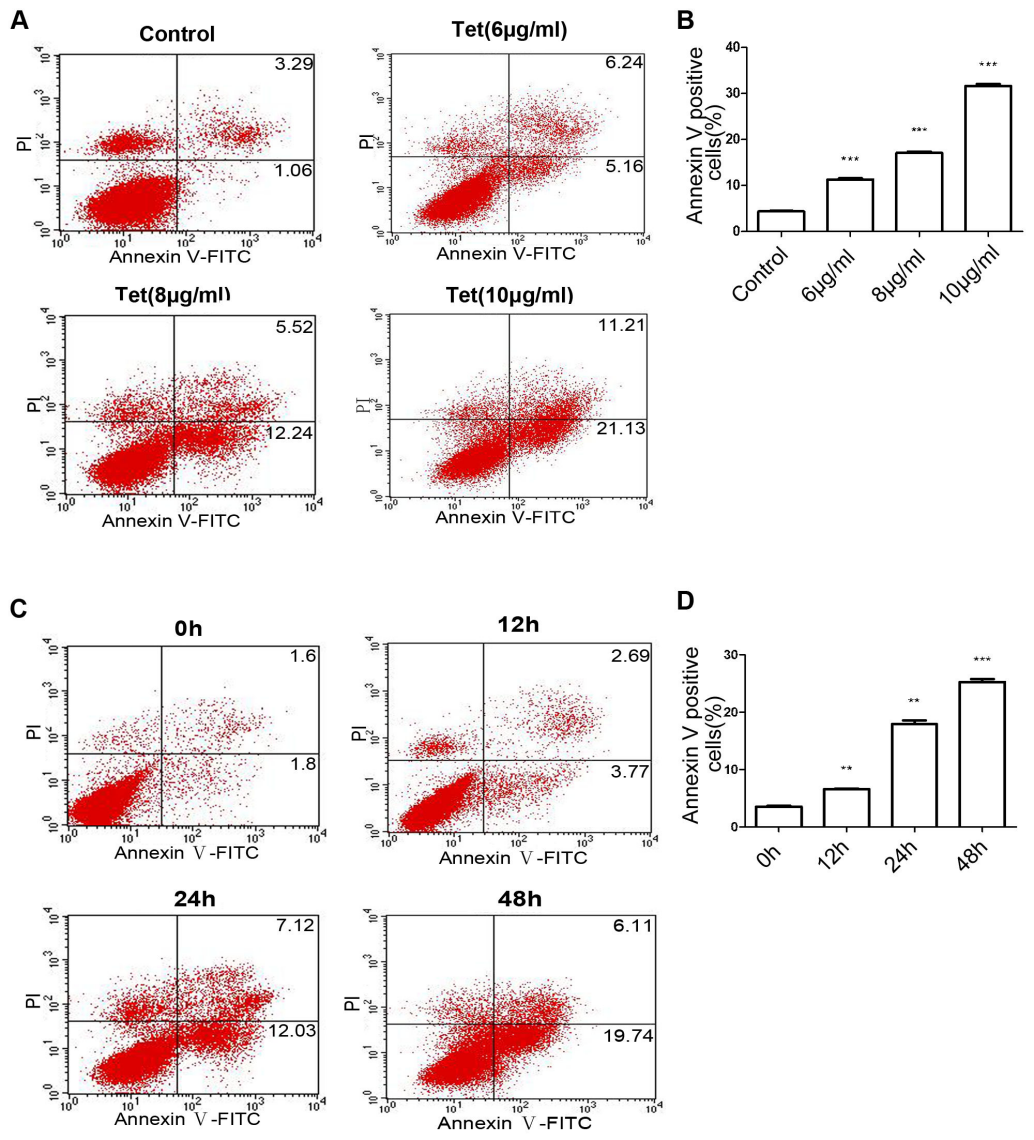
Apoptosis of BGC-823 cells induced by tetrandrine. A PI-Annexin V-FITC binding assay was used to detect apoptosis of BGC-823 cells. (A) Cells treated with tetrandrine for 24 h at 0, 6, 8, and 10 μg/ml. (C) Cells treated with 8 μg/ml tetrandrine for 0, 12, 24, and 48 h. (B, D) Columns represent the means ± SD of apoptotic cells obtained from three independent experiments. **P < 0.01; ***P < 0.001 versus the control group.

Tetrandrine-mediated anti-cancer abilities have been reported to be associated with apoptosis [9-12]. Accordingly, we investigated the ability of tetrandrine-induced apoptosis of BGC-823 cells using a flow cytometry assay. As measured by flow cytometry, the effects of tetrandrine on BGC-823 cell apoptosis are shown in Figure 2A and B, with the 24-h tetrandrine treatments at 0, 6, 8, and 10 μg/ml resulting in 4.35%, 11.4%, 17.72%, and 32.34% of apoptotic cells, respectively, and tetrandrine (8 μg/ml) for 0, 12, 24, and 48 h resulting in 3.4%, 6.64%, 19.15%, and 25.85% of apoptotic cells, respectively (Figure 2C and D). These data clearly indicated that tetrandrine induced apoptosis in BGC-823 cells in a dose- and time-dependent manner. The numbers of apoptotic cells increased along with the tetrandrine concentration and treatment time.

### Tetrandrine-mediated expression of Bcl-2 family members

**Figure 3:**
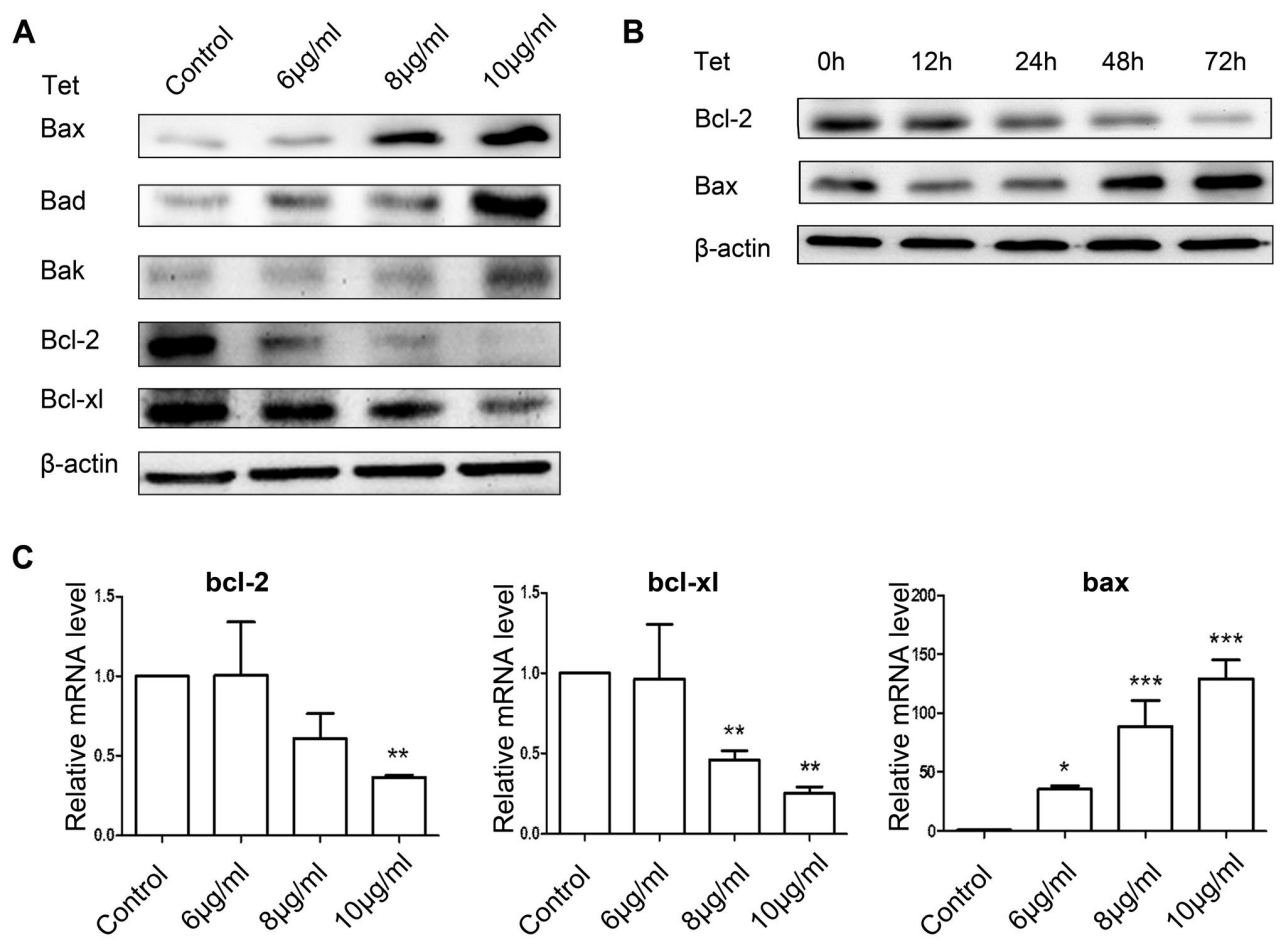
The effect of tetrandrine on apoptosis-related proteins in BGC-823 cells. (A) Western blot analysis of the expression of apoptosis-related proteins in BGC-823 cells treated with 6, 8, and 10 μg/ml tetrandrine for 24 h. (B) Western blot analysis of bcl-2 and bax treated with 8 μg/ml tetrandrine for 12, 24, 48, and 72 h. (C) Effects of tetrandrine on the expression levels of bcl-2, bcl-xl, and bax were determined using real-time PCR (1), control (2) 6 μg/ml (3) 8 μg/ml (4) 10 μg/ml. β-actin expression was used as an internal control. Data are reported as the means ± SD of at least three experiments. *P < 0.05; **P < 0.01; ***P < 0.001 versus the control group.

To investigate the more specific mechanism on the induction of apoptosis in BGC-823 cells by tetrandrine, we detected the expression of apoptosis-related proteins by Western blotting and their mRNA level by real-time (RT)-PCR. Here, we studied the expression of Bax, Bad, Bak, Bcl-2, and Bcl-xl in BGC-823 cells treated with tetrandrine. After 24 h of exposure to tetrandrine, Bax, Bad, and Bak protein expression was dose-dependently increased, whereas Bcl-2 and Bcl-xl protein expression was dose-dependently decreased (Figure 3A). We also found that tetrandrine could upregulate the expression of Bax and downregulate the expression of Bcl-2 in a time-dependent manner (Figure 3B). To determine whether tetrandrine would affect the gene transcription of bcl-2, bcl-xl, and bax, their mRNA expression was examined by RT-PCR (Figure 3C). The RT-PCR results clearly coincided with those presented in Figure 3A.

### Tetrandrine-induced apoptosis via a mitochondrial pathway in BGC-823 cells

**Figure 4:**
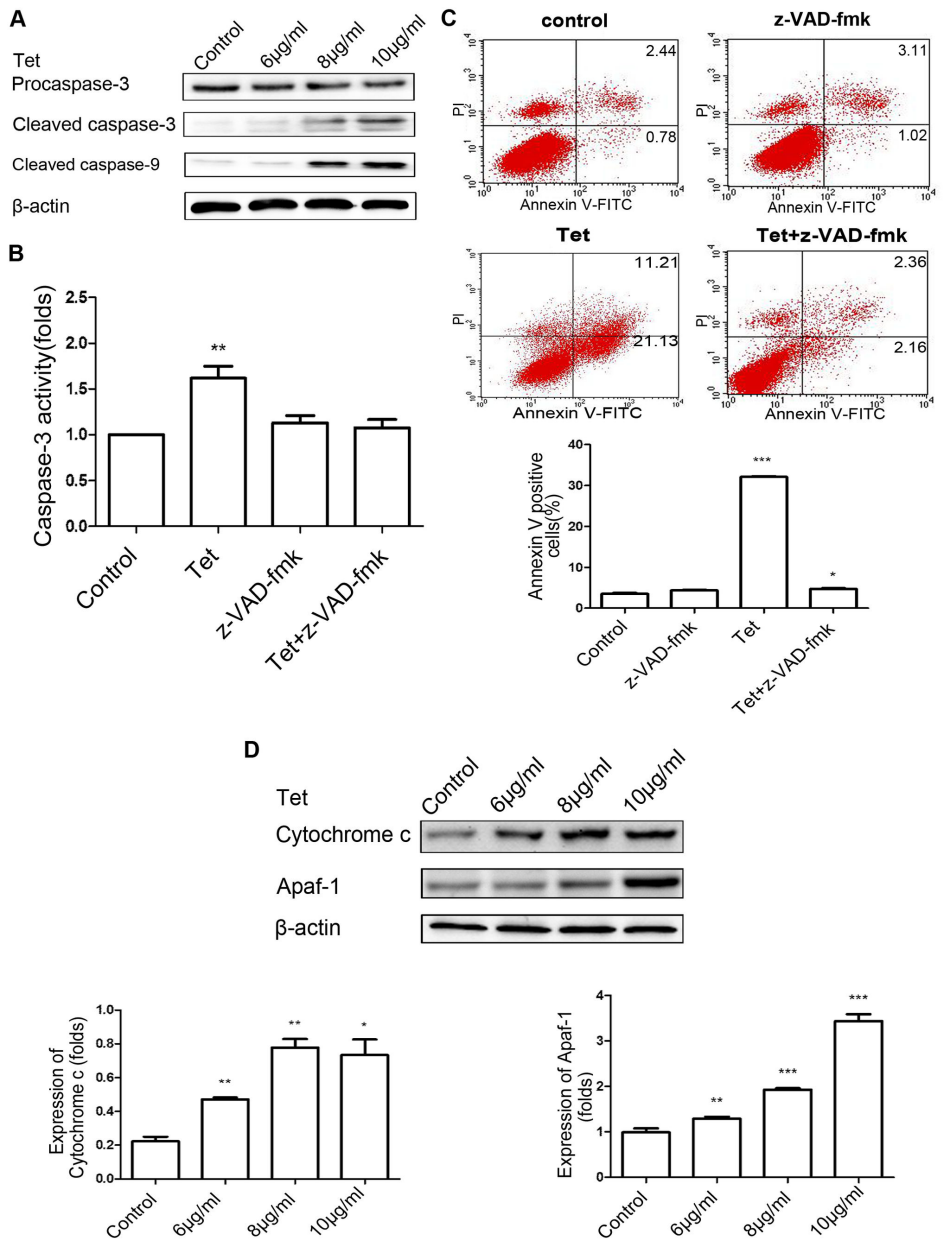
Tetrandrine-induced apoptosis via a mitochondrial pathway in BGC-823 cells. (A) Western blot analysis of the expression of procaspase-3, cleaved caspase-3, and cleaved caspase-9 in BGC-823 cells treated with tetrandrine at 6, 8, and 10 μg/ml for 24 h. (B) The cells were pretreated with or without pan-caspase inhibitor z-VAD-fmk (10 μM) for 4 h followed by treatment with tetrandrine at 8 μg/ml for 24 h, and the relative activity of caspase-3 was measured using a caspase-3 activity assay. (C) BGC-823 cells were pretreated with or without z-VAD-fmk (10 μM) for 4 h followed by treatment with tetrandrine at 8 μg/ml for 24 h. A PI-Annexin V-FITC binding assay was used to detect apoptosis of BGC-823 cells. (D) Western blot analysis of the expression of cytoplasmic cytochrome *c* and Apaf-1 in BGC-823 cells treated with tetrandrine at 6, 8, and 10 μg/ml for 24 h. β-actin expression was used as an internal control. Data are reported as the means ± SD of at least three experiments. *P < 0.05; **P < 0.01; ***P < 0.001 versus the control group.

To explore whether tetrandrine-induced apoptosis was associated with the activation of caspase members, we measured the expression of caspases in BGC-823 cells treated with various concentrations of tetrandrine by Western blotting. The results showed a dose-dependent elevation of cleaved caspase-3 and cleaved caspase-9 in tetrandrine- treated cells (Figure 4A). However, nondetectable levels of cleaved caspase-3 and cleaved caspase-9 were found in the cells in the absence of tetrandrine treatment (data not shown). To further determine the role of caspase activation in tetrandrine-induced apoptosis, BGC-823 cells were pretreated with the pan-caspase inhibitor z-VAD-fmk (10 μM) for 4 h, and then treated with 8 μg/ml tetrandrine for 24 h. Compared to the control, the relative activities of caspase-3 were increased in BGC-823 cells treated with tetrandrine only (Figure 4B). In addition, the release of cytochrome *c* was increased in a dose-dependent manner by tetrandrine. A representative flow cytometry result showed that cells pretreated with z-VAD-fmk strongly reduced tetrandrine-induced apoptosis and that only a small number of apoptotic cells was detected (Figure 4C). Next, we examined the expression level of cytoplasmic cytochrome *c*, which is released from the mitochondria into the cytoplasm during apoptosis, acted on apoptotic protease activating factor-1 (Apaf-1) by increasing the binding of Apaf-1 to ATP/dATP, and induced caspase-9-dependent activation of caspase-3 [20-22]. It was found that tetrandrine significantly increased the expression of cytoplasmic cytochrome *c* and Apaf-1 in BGC-823 cells in a dose-dependent manner (Figure 4D). Together, these findings suggested that a mitochondria-mediated caspase cascade pathway was involved in tetrandrine-induced apoptosis.

### Anti-tumor effect of tetrandrine on BGC-823 nude mouse xenografts

**Figure 5:**
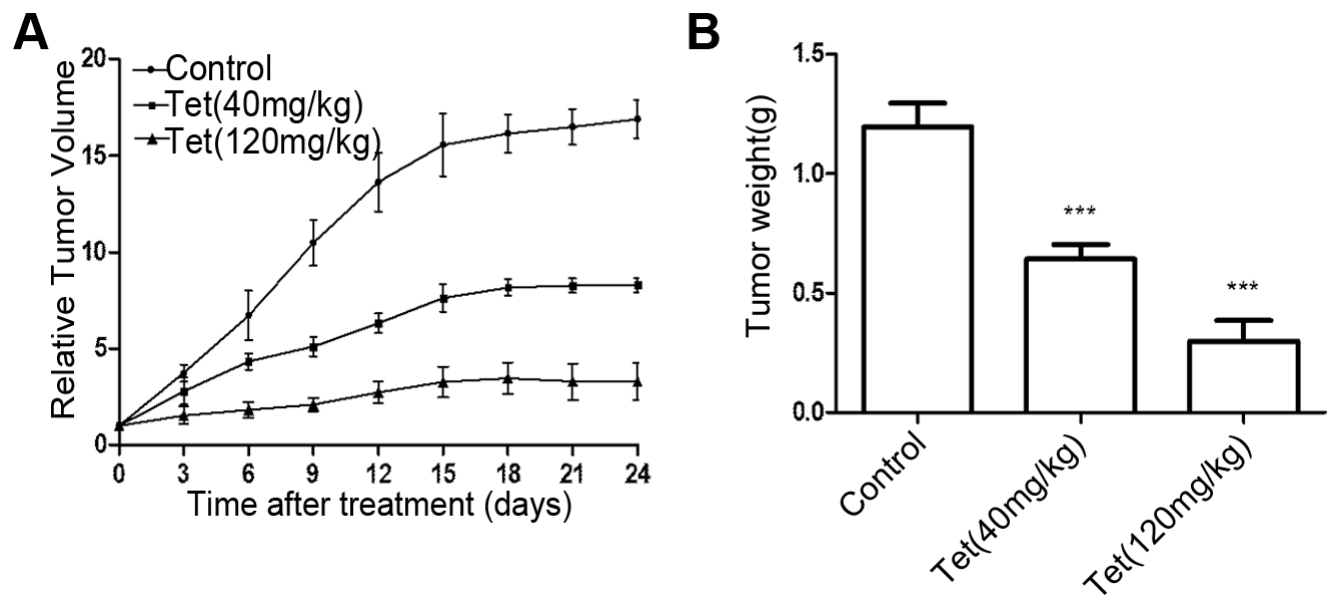
Tetrandrine inhibits tumor growth in vivo. BGC-823 cells were subcutaneously inoculated into BALB/c nude mice to establish the xenograft model. (A) Tumor growth was monitored at the indicated time points, and the tumor volume was measured every 3 days. The starting day of drug treatment was defined as day 0. Data represent means ± SD of the relative tumor volume for each group. (B) Tumor weight was measured at the end of the experiment. Data represent means ± SD of the tumor weight for each group. ***P < 0.001 versus the control group.

Since tetrandrine was found to significantly inhibit the growth of BGC-823 cells *in vitro*, we further evaluated the anti-tumor effect of tetrandrine *in vivo* with established xenograft models. As shown in Figure 5A, tetrandrine could effectively inhibit tumor growth after a 24-day treatment. At the end of 24 days, the average tumor volumes were 493.06 mm^3^ and 1243.00 mm^3^ in the high dose group and low dose group, respectively, corresponding to 80.52% and 50.9% inhibition rate compared to the control group. In addition, tumor weight was reduced as a result of treatment with tetrandrine at both concentrations (Figure 5B & Table 2). This result showed that a high dose of tetrandrine had a stronger anti-tumor effect than a low dose on the BGC-823 xenograft model.

**Table 2:** Inhibition of tetrandrine (Tet) on tumor growth of nude mice.

**Group (n = 5)**	**Tumor volume(mm^3^)**	**Inhibitory Rate (%)**	**Tumor weight (g)**	**Inhibitory Rate (%)**
control	2531.78 ± 153.25		1.194 ± 0.10	
Tet (40 mg/kg)	1243.00 ± 55.19^***^	50.90	0.641 ± 0.06^***^	46.31
Tet(120 mg/kg)	493.06 ± 145.63^***^	80.52	0.298 ± 0.09^***^	75.04

^***^ *P* < 0.001 *versus* the control group.

### Tetrandrine-induced apoptosis in BGC-823 xenografts

**Figure 6:**
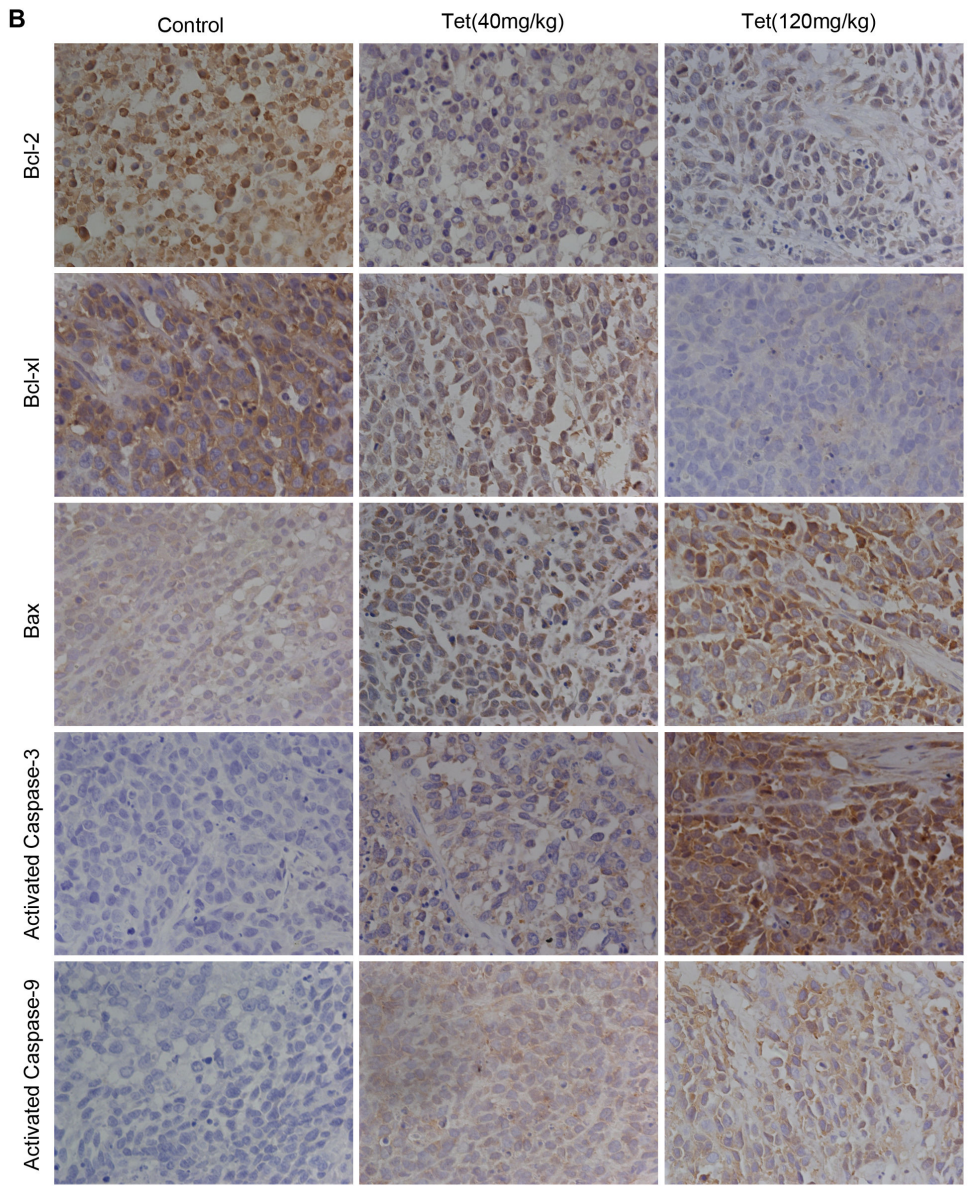
Immunohistochemical staining for apoptosis-related protein in BGC-823 xenografts. Immunohistochemical staining for apoptosis-related proteins: Bcl-2, Bcl-xl, Bax, Activated caspase-3, and Activated capase-9. Magnification is 200×. Representative microphotographs of three groups are shown. DAB stained immunoreactive cells (dark brown).

Immunohistochemistry analysis of tumor tissues excised from BGC-823 xenografted nude mice reconfirmed that apoptosis had occurred in tetrandrine-treated tumors and that the mitochondrial pathway played a significant role during apoptosis. There were five nude mice in each group. We observed that the expression of Bcl-2 and Bcl-xl was decreased, whereas the protein levels of Bax, activated caspase-3, and activated caspase-9 were significantly enhanced by tetrandrine (Figure 6). All these findings strongly corroborated that tetrandrine may induce apoptosis, consistent with the *in vitro* study.

## Discussion

Gastric cancer, the fourth most commonly diagnosed neoplasm behind cancer of the lung, breast, colon, and rectum, causes nearly one million annual deaths and has been the second cause of cancer-related mortality worldwide [23-26]. Surgery, chemoradiotherapy, and molecular targeted therapy have been the main treatment strategies in the treatment of gastric cancer [27]. Although surgical resection has been the most effective curative treatment, the results are inconsistent and limited by tumor recurrence and chemoresistance. Conventional chemotherapeutic drugs, such as Ptx, 5-fluorouracil, and cisplatin kill tumor cells by inducing apoptosis and autophagy; but tumor cells may become resistant to drug-induced apoptosis [17,28,29].

It is reported that tetrandrine has strong anti-tumor activity both *in vitro* and *in vivo* in many cancer cells, such as A549 human lung carcinoma cells, bladder cancer cells, colon cancer cells, hepatoma cells, and so on [9-12]. A previous study indicated that tetrandrine might play a promising role by synergistic interaction with chemotherapeutic agents possibly by their synergistic effects on inducing apoptosis and downregulating chemotherapeutic agent-associated genes in MKN-28 cells and BGC-823 cells [18]. However, the mechanism of tetrandrine on human gastric cancer BGC-823 cells has not been identified. In this study, we demonstrated that tetrandrine exhibited potent antitumor activity toward human gastric cancer *in vitro* and *in vivo*, elucidating the underlying mechanisms of action.

Firstly, our results showed that tetrandrine effectively inhibited BGC-823 cell proliferation assayed by MTT in a dose- and time-dependent manner. Meanwhile, the IC_50_s of tetrandrine were approximately 6.1, 4.5, and 3.7 μg/ml in BGC823 cells for 24, 48, and 72 h, respectively. According to previous studies, chemotherapeutic drugs kill solid tumors mainly by inducing apoptosis [30-32]. We found that apoptosis was induced by tetrandrine in a concentration-dependent manner through flow cytometry analysis with increased early apoptotic cells and late apoptotic cells in gastric cancer BGC-823 cells.

In mammalian cells, apoptosis can be triggered by the extrinsic pathway activated by the death receptor and the intrinsic pathway regulated by Bcl-2 family members and caspase cascades in the mitochondrion [33,34]. In our study, tetrandrine increased the expression of the pro-apoptotic proteins Bax, Bad, and Bak and decreased the protein levels of Bcl-2 and Bcl-xl. Therefore, an increased ratio of anti- and pro-apoptotic protein expression, such as the Bax/Bcl-2 ratio, would lead to the loss of mitochondrial membrane potential. Dysfunctioning mitochondria releases cytochrome *c*, which accumulates in the cytosol and forms a complex with apaf-1. In the intrinsic pathway, as an effector, caspase-3 is cleaved by activation of caspase-9 [35-37]. In the present study, we showed that caspase-9 and caspase-3 were activated by tetrandrine. The pan-caspase inhibitor z-VAD-fmk reduced apoptosis in BGC-823 cells, indicating that the mitochondrial pathway plays a central role in tetrandrine-induced apoptosis.

It is reported that tetrandrine could reverse the resistance to many chemotherapy drugs, such as Ptx and doxorubicin in the KBv200 and K562 nude mouse xenograft models [38,39]. Tetrandrine also possesses a strong ability to inhibit angiogenesis in gliomas in rats [40]. Furthermore, our results indicated that tetrandrine exhibited strong anti-tumor activity by inhibiting tumor growth and inducing apoptosis in the established BGC-823 nude mouse xenograft model.

In conclusion, our data demonstrated that tetrandrine significantly induced apoptosis in human gastric cancer BGC-823 cells and its nude mouse xenograft models. Tetrandrine-induced apoptosis was associated with activation of the intrinsic apoptosis pathway. Based on these results, tetrandrine could have great potential in the treatment of human gastric cancer in the future.

## References

[1] Yang L (2006) Incidence and mortality of gastric cancer in China. World J Gastroenterol 12: 17-20. PubMed: 16440411. 16440411 10.3748/wjg.v12.i1.17 PMC4077485

[2] Leung WK, Wu MS, Kakugawa Y, Kim JJ, Yeoh KG et al. (2008) Screening for gastric cancer in Asia: current evidence and practice. Lancet Oncol 9: 279-287. doi:10.1016/S1470-2045(08)70072-X. PubMed: 18308253. 18308253 10.1016/S1470-2045(08)70072-X

[3] Vogiatzi P, Vindigni C, Roviello F, Renieri A, Giordano A (2004) Deciphering the underlying genetic and epigenetic events leading to gastric carcinogenesis. J Cell Physiol 211: 287-295. PubMed: 17238139. 10.1002/jcp.20982 17238139

[4] Lochhead P, El-Omar EM (2008) Gastric cancer. Br Med Bull 85: 87-100. doi:10.1093/bmb/ldn007. PubMed: 18267927. 18267927 10.1093/bmb/ldn007

[5] Meyer HJ, Wilke H (2011) Treatment strategies in gastric cancer. Deut Arzteblatt Int 108: 698-706. PubMed: 22114638. 10.3238/arztebl.2011.0698 PMC3221435 22114638

[6] Zhang L, Ji Q, Ni ZH, Sun J (2012) Prohibitin Induces Apoptosis in BGC823 Gastric Cancer Cells through the Mitochondrial Pathway. Asian Pac J Cancer Prev 13: 3803-3807. doi:10.7314/APJCP.2012.13.8.3803. PubMed: 23098474. 23098474 10.7314/apjcp.2012.13.8.3803

[7] Pang L, Hoult JR (1997) Cytotoxicity to macrophages of tetrandrine, an antisilicosis alkaloid, accompanied by an overproduction of prostaglandins. Biochem Pharmacol 53: 773-782. doi:10.1016/S0006-2952(96)00817-9. PubMed: 9113098. 9113098 10.1016/s0006-2952(96)00817-9

[8] Chen B, Sun Q, Wang X, Gao F, Dai Y et al. (2008) Reversal in multidrug resistance by magnetic nanoparticle of Fe_3_O_4_ loaded with adriamycin and tetrandrine in K562/A02 leukemic cells. Int J Nanomed 3: 277-286. PubMed: 18686787. 10.2147/ijn.s2714 PMC2527663 18686787

[9] Cho HS, Chang SH, Chung YS, Gao F, Dai Y et al. (2009) Synergistic effect of ERK inhibition on tetrandrine-induced apoptosis in A549 human lung carcinoma cells. J Vet Sci 10: 23-28. doi:10.4142/jvs.2009.10.1.23. PubMed: 19255520. 19255520 10.4142/jvs.2009.10.1.23 PMC2801106

[10] Li X, Su B, Liu R, Wu D, He D (2011) Tetrandrine induces apoptosis and triggers caspase cascade in human bladder cancer cells. J Surg Res 166: e45-e51. doi:10.1016/j.jss.2010.10.034. PubMed: 21176918. 21176918 10.1016/j.jss.2010.10.034

[11] Wu JM, Chen Y, Chen JC, Lin TY, Tseng SH (2010) Tetrandrine induces apoptosis and growth suppression of colon cancer cells in mice. Cancer Lett 287: 187-195. doi:10.1016/j.canlet.2009.06.009. PubMed: 19586712. 19586712 10.1016/j.canlet.2009.06.009

[12] Ng LT, Chiang LC, Lin YT, Lin CC (2006) Antiproliferative and apoptotic effects of tetrandrine on different human hepatoma cell lines. Am J Chin Med 34: 125-135. doi:10.1142/S0192415X06003692. PubMed: 16437745. 16437745 10.1142/S0192415X06003692

[13] Jang BC, Lim KJ, Paik JH, Cho JW, Baek WK et al. (2004) Tetrandrine-induced apoptosis is mediated by activation of caspases and PKC-delta in U937 cells. Biochem Pharmacol 67: 1819-1829. doi:10.1016/j.bcp.2004.01.018. PubMed: 15130759. 15130759 10.1016/j.bcp.2004.01.018

[14] Li X, Lu X, Xu H, Zhu Z, Yin H et al. (2012) Paclitaxel/tetrandrine coloaded nanoparticles effectively promote the apoptosis of gastric cancer cells based on "oxidation therapy". Mol Pharm 9: 222-229. doi:10.1021/mp2002736. PubMed: 22171565. 22171565 10.1021/mp2002736

[15] Dorr RT (1994) Pharmacology and toxicology of Cremophor EL diluents. Ann Pharmacother 28: S11-S14. PubMed: 7915152. 7915152 10.1177/10600280940280S503

[16] Bissery MC, Nohynek G, Sanderink GJ, Lavelle F (1995) Docetaxel (Taxotere): a review of preclinical and clinical experience. Part I: Preclinical experience. Anti Cancer Drugs 6: 339-368. 7670132 10.1097/00001813-199506000-00001

[17] Tabuchi Y, Matsuoka J, Gunduz M, Imada T, Ono R et al. (2009) Resistance to paclitaxel therapy is related with Bcl-2 expression through an estrogen receptor mediated pathway in breast cancer. Int J Oncol 34: 313-319. PubMed: 19148464. 19148464

[18] Wei J, Liu B, Wang L, Qian X, Ding Y et al. (2007) Synergistic interaction between tetrandrine and chemotherapeutic agents and influence of tetrandrine on chemotherapeutic agent-associated genes in human gastric cancer cell lines. Cancer Chemother Pharmacol 60: 703-711. doi:10.1007/s00280-007-0416-9. PubMed: 17256130. 17256130 10.1007/s00280-007-0416-9

[19] Wei N, Liu GT, Chen XG, Liu Q, Wang FP et al. (2011) H1, a derivative of Tetrandrine, exerts anti-MDR activity by initiating intrinsic apoptosis pathway and inhibiting the activation of Erk1/2 and Akt1/2. Biochem Pharmacol 82: 1593-1603. doi:10.1016/j.bcp.2011.08.012. PubMed: 21864508. 21864508 10.1016/j.bcp.2011.08.012

[20] Wang C, Youle RJ (2009) The role of mitochondria in apoptosis. Annu Rev Genet 43: 95-118. doi:10.1146/annurev-genet-102108-134850. PubMed: 19659442. 19659442 10.1146/annurev-genet-102108-134850 PMC4762029

[21] Luo X, Budihardjo I, Zou H, Slaughter C, Wang X (1998) Bid, a Bcl2 interacting protein, mediates cytochrome c release from mitochondria in response to activation of cell surface death receptors. Cell 94: 481-490. doi:10.1016/S0092-8674(00)81589-5. PubMed: 9727491. 9727491 10.1016/s0092-8674(00)81589-5

[22] Atsumi T, Kato K, Uno K, Iijima K, Koike T et al. (2007) Pathophysiological role of the activation of p38 mitogen-activated protein kinases in poorly differentiated gastric cancer. Pathol Int 57: 635-644. doi:10.1111/j.1440-1827.2007.02152.x. PubMed: 17803652. 17803652 10.1111/j.1440-1827.2007.02152.x

[23] Parkin DM, Bray FI, Devesa SS (2001) Cancer burden in the year 2000. The global picture. Eur J Cancer 37: S4-S66. doi:10.1016/S0959-8049(01)80044-7. PubMed: 11602373. 11602373 10.1016/s0959-8049(01)00267-2

[24] Parkin DM (2004) International variation. Oncogene 23: 6329-6340. doi:10.1038/sj.onc.1207726. PubMed: 15322508. 15322508 10.1038/sj.onc.1207726

[25] Ferlay J, Shin HR, Bray F, Forman D, Mathers C et al. (2010) Estimates of worldwide burden of cancer in 2008: GLOBOCAN 2008. Int J Cancer 127: 2893-2917. doi:10.1002/ijc.25516. PubMed: 21351269. 21351269 10.1002/ijc.25516

[26] Ford AC (2011). Best practice & research. Clinical gastroenterology 25: 581-592. doi:10.1016/j.bpg.2011.09.002. PubMed: 22122773. 22122773 10.1016/j.bpg.2011.09.002

[27] Roukos DH, Kappas AM (2005). Nat Clin Practice Oncol 2: 98-107. doi:10.1038/ncponc0099. 10.1038/ncponc0099 16264882

[28] Jiang M, Wang CY, Huang S, Yang T, Dong Z (2009) Cisplatin-induced apoptosis in p53-deficient renal cells via the intrinsic mitochondrial pathway. Am J Physiol Renal Physiol 296: F983-F993. doi:10.1152/ajprenal.90579.2008. PubMed: 19279129. 19279129 10.1152/ajprenal.90579.2008 PMC2681364

[29] Wu Y, Shen D, Chen Z, Clayton S, Vadgama JV (2007) Taxol induced apoptosis regulates amino acid transport in breast cancer cells. Apoptosis 12: 593–612. doi:10.1007/s10495-006-0007-y. PubMed: 17195090. 17195090 10.1007/s10495-006-0007-y

[30] Gu WJ, Liu HL (2013) Induction of pancreatic cancer cell apoptosis, invasion, migration, and enhancement of chemotherapy sensitivity of gemcitabine, 5-FU, and oxaliplatin by hnRNP A2/B1 siRNA. Anti Cancer Drugs 24(6):566-76. PubMed: 23525071. 23525071 10.1097/CAD.0b013e3283608bc5

[31] Pan X, Zhang X, Sun H, Zhang J, Yan M et al. (2013) Autophagy inhibition promotes 5-fluorouraci-induced apoptosis by stimulating ROS formation in human non-small cell lung cancer A549 cells. PLOS ONE 8: e56679. doi:10.1371/journal.pone.0056679. PubMed: 23441212. 23441212 10.1371/journal.pone.0056679 PMC3575481

[32] Rogalska A, Marczak A, Gajek A, Szwed M, Śliwińska A et al. (2013) Induction of apoptosis in human ovarian cancer cells by new anticancer compounds, epothilone A and B. Toxicol In Vitro 27:239-49. 22995584 10.1016/j.tiv.2012.09.006

[33] Fulda S, Debatin KM (2006) Extrinsic versus intrinsic apoptosis pathways in anticancer chemotherapy. Oncogene 25: 4798-4811. doi:10.1038/sj.onc.1209608. PubMed: 16892092. 16892092 10.1038/sj.onc.1209608

[34] Hirsch T, Marzo I, Kroemer G (1997) Role of the mitochondrial permeability transition pore in apoptosis. Biosci Rep 17: 67-76. doi:10.1023/A:1027339418683. PubMed: 9171922. 9171922 10.1023/a:1027339418683

[35] Susin SA, Daugas E, Ravagnan L, Samejima K, Zamzami N et al. (2000) Two distinct pathways leading to nuclear apoptosis. J Exp Med 192: 571-580. doi:10.1084/jem.192.4.571. PubMed: 10952727. 10952727 10.1084/jem.192.4.571 PMC2193229

[36] Slee EA, Harte MT, Kluck RM, Wolf BB, Casiano CA et al. (1999) Ordering the cytochrome c-initiated caspase cascade: hierarchical activation of caspases-2, -3, -6, -7, -8, and -10 in a caspase-9-dependent manner. J Cell Biol 144: 281-292. doi:10.1083/jcb.144.2.281. PubMed: 9922454. 9922454 10.1083/jcb.144.2.281 PMC2132895

[37] Lee HJ, Lee HJ, Lee EO, Ko SG, Bae HS et al. (2008) Mitochondria-cytochrome C-caspase-9 cascade mediates isorhamnetin-induced apoptosis. Cancer Lett 270: 342-353. doi:10.1016/j.canlet.2008.05.040. PubMed: 18617322. 18617322 10.1016/j.canlet.2008.05.040

[38] Dai CL, Xiong HY, Tang LF, Zhang X, Liang YJ et al. (2007) Tetrandrine achieved plasma concentrations capable of reversing MDR in vitro and had no apparent effect on doxorubicin pharmacokinetics in mice. Cancer Chemother Pharmacol 60: 741-750. doi:10.1007/s00280-007-0420-0. PubMed: 17273824. 17273824 10.1007/s00280-007-0420-0

[39] Zhu XM, Sui MH, Fan WM (2005) In vitro and in vivo characterizations of tetrandrine on the reversal of P-glycoprotein-mediated drug resistance to paclitaxel. Anticancer Res 25: 1953-1962. PubMed: 16158930. 16158930

[40] Chen Y, Chen JC, Tseng SH (2009) Tetrandrine suppresses tumor growth and angiogenesis of gliomas in rats. Int J Cancer 124: 2260-2269. doi:10.1002/ijc.24208. PubMed: 19165864. 19165864 10.1002/ijc.24208

